# Genetic Variation and Exchange in *Trypanosoma cruzi* Isolates from the United States

**DOI:** 10.1371/journal.pone.0056198

**Published:** 2013-02-14

**Authors:** Dawn M. Roellig, Mason Y. Savage, A. Wendy Fujita, Christian Barnabé, Michel Tibayrenc, Frank J. Steurer, Michael J. Yabsley

**Affiliations:** 1 Southeastern Cooperative Wildlife Disease Study, College of Veterinary Medicine, University of Georgia, Athens, Georgia, United States of America; 2 Department of Infectious Diseases, College of Veterinary Medicine, University of Georgia, Athens, Georgia, United States of America; 3 MIVEGEC (Université de Montpellier 1 et 2 - CNRS 5290 - IRD 224), Maladies Infectieuses et Vecteurs : Ecologie, Génétique, Evolution et Contrôle, Institut de Recherche pour le Développement (IRD), Representation in Bolivia, La Paz, Bolivia; 4 MIVEGEC (Université de Montpellier 1 et 2 - CNRS 5290 - IRD 224), Maladies Infectieuses et Vecteurs : Ecologie, Génétique, Evolution et Contrôle, Institut de Recherche pour le Développement/Université Montpellier I/Centre National de la Recherche Scientifique, IRD Center, Montpellier, France; 5 Centers for Disease Control and Prevention, Atlanta, Georgia, United States of America; 6 D.B. Warnell School of Forestry and Natural Resources, University of Georgia, Athens, Georgia, United States of America; Federal University of São Paulo, Brazil

## Abstract

*Trypanosoma cruzi*, the causative agent of Chagas disease, is a multiclonal parasite with high levels of genetic diversity and broad host and geographic ranges. Molecular characterization of South American isolates of *T. cruzi* has demonstrated homologous recombination and nuclear hybridization, as well as the presence of 6 main genetic clusters or “discrete typing units” (DTUs). Few studies have extensively investigated such exchange events and genetic diversity in North American isolates. In the current study, we genetically characterized over 50 US isolates from wildlife reservoirs (e.g., raccoons, opossums, armadillos, skunks), domestic dogs, humans, nonhuman primates, and reduviid vectors from nine states (TX, CA, OK, SC, FL, GA, MD, LA, TN) using a multilocus sequencing method. Single nucleotide polymorphisms were identified in sequences of the mismatch-repair class 2 (*MSH2*) and *Tc52* genes. Typing based on the two genes often paralleled genotyping by classic methodologies using mini-exon and 18S and 24Sα rRNA genes. Evidence for genetic exchange was obtained by comparing sequence phylogenies of nuclear and mitochondrial gene targets, dihydrofolate reductase-thymidylate synthase (*DHFR-TS*) and the cytochrome oxidase subunit II- NADH dehydrogenase subunit I region (*COII-ND1*), respectively. We observed genetic exchange in several US isolates as demonstrated by incongruent mitochondrial and nuclear genes phylogenies, which confirms a previous finding of a single genetic exchange event in a Florida isolate. The presence of SNPs and evidence of genetic exchange illustrates that strains from the US are genetically diverse, even though only two phylogenetic lineages have been identified in this region.

## Introduction

*Trypanosoma cruzi*, the causative agent of Chagas disease, is a clonally proliferative parasite with a heterogeneous population [1], [2]. It is a biologically, molecularly, and biochemically diverse parasite that has been detected in over 200 mammalian species, including humans [3]. Prior to advances in molecular biology and genetics, differences in *T. cruzi* were based solely on growth characteristics and manifestations of disease in various hosts [4]. Today, *T. cruzi* is segregated into six major discrete typing units, TcI to TcVI and a scarcely described Tcbat genotype [5]–[8]. Characterizing a strain of *T. cruzi* into one of these six genotypes is useful in determining the evolutionary ecology of the parasite in a region, as well as, associating biological characters with disease manifestations. Previously, a predominately clonal population structure for *T. cruzi* was accepted [9], but with recent evidence for genetic exchange events, hybridization, and mitochondrial introgression this model has been challenged [10]–[17].

Looking at ten intergenic regions of the *T. cruzi* genome in well-characterized isolates, TcV and TcVI have been confirmed as direct hybrids of parental groups TcII and TcIII [18]. Machado and Ayala [10] have also illustrated genetic exchange by comparing nuclear and mitochondrial gene phylogenies and while the majority of isolates included in their study were from South and Central America, three isolates were from the US, of which one exhibited genetic exchange. Additionally, experimental evidence of genetic exchange in a laboratory system was revealed with the hybridization of clones [11]; however, such events in nature are rare [9] and the mechanism of recombination in *T. cruzi* is still unknown.

Because *T. cruzi* is such a significant cause of morbidity and mortality in Central and South America, considerable characterization work on *T. cruzi* has been conducted in these regions, but because human cases in the US are rare, little work has been conducted to characterize US isolates. Since 1955, six autochthonous human cases have been reported in the United States with the most recent occurring in 2006 [19]–[23]. In addition to these six cases, over 1,750 individual blood donors currently residing in the United States were positive for antibodies reactive to *T. cruzi* [24]. The objective of this study was to explore the molecular diversity of *T. cruzi* from the US. Our goals were to investigate genetic exchange in US isolates and compare sequences of several gene targets with those from South America to identify evidence of genetic diversity between these different regions. To accomplish these goals, nucleotide sequences of two nuclear genes, the mismatch-repair class 2 gene (*MSH2*) and the thiol-disulfide oxido-reductase Tc52 gene (*Tc52*), were compared with a selection of *T. cruzi* isolates from the United States to identify single nucleotide polymorphisms that indicate heterogeneity and potential virulence differences. To investigate potential genetic exchange, the phylogenies of a nuclear gene [dihydrofolate reductase-thymidylate synthase (*DHFR-TS*)] and mitochondrial gene targets [cytochrome oxidase subunit II-NADH dehydrogenase subunit I region (*COII-ND1*)] were compared.

## Materials and Methods

### Ethics Statement

Samples labeled as “human” origin and from various animals were obtained through a material transfer agreement with CDC, Pasteur Institute, and Southeastern Cooperative Wildlife Disease Study. The conditions and approvals for the archived samples are unknown to the authors. No authors came in contact with human subjects during the research. Remaining samples from animals were obtained by handling wild-trapped animals. These animals were cared for in accordance with the guidelines of the Institutional Animal Care and use Committee and under animal use protocol A2009-3-006 approved by this committee at the University of Georgia.

### Isolates

*T. cruzi* was isolated from multiple species of free-ranging and captive wildlife, domestic animals, triatomine bug vectors, and humans who were autochthonously infected in the United States; host and origin of each isolate can be seen in Table 1. Some isolates were obtained as liquid nitrogen-stored parasites from the Centers for Disease Control and Prevention, Pasteur Institute, and the Southeastern Cooperative Wildlife Disease Study and were established in axenic LIT medium as previously described [25]. Additional isolates were obtained from wild-trapped animals in axenic LIT medium or canine macrophage-cell culture as previously described [26]. Biological clones are indicated with the prefix “clX.”

**Table 1:** Lineage typing of *Trypanosoma cruzi* isolates from the United States.

				Gene target			
Host (Order)	Isolate	Origin	Lineage>>†	Tc52	MSH2	DHFR-TS	COII-ND1
Human	TC CC	Corpus Christi, TX	I	I	I	I‡	n.d.
(Primate)	CA R	California	I	I	I	I	I
	TC California	Lake Don Pedro, TX	I	I	I	n.d.	I‡
Domestic Dog	Caesar Dog	Not known	IV	IV	IV	IV	IV
(Carnivora)	Dog Theis	Not known	IV	IV	IV	IV	IV
	Griffin Dog	Hillsboro, TN	I/IV	IV/I	IV/I	IV	I
	OK Dog	Bartlesville, OK	IV	IV	IV	IV	IV
	Samantha Dog	South Carolina	IV	IV	IV	IV	IV
	Smokey	South Carolina	IV	IV	IV	IV	IV‡
	USA Dog Y	California	IV	I	I	I	IV
VA Opossum	92101601P cl2	Statesboro, GA	n.d.	I	I	I	IV
(Didelphimorphia)	93041401P cl1	Statesboro, GA	I	I	I	I	IV‡
	93070103P cl2	Fort Stewart, GA	I	I	I	I	IV
	FH4	South Georgia	I	I	I	I	IV
	FL Opo 2	Wakulla Springs, FL	I	I	I	I	IV
	FL Opo 3	Wakulla Springs, FL	I	I	I	I	IV
	FL Opo 15	Maclay State Park, FL	I	I	I	n.d.	IV
	Opossum 1970	New Orleans, LA	I	I	I	I	I‡
	USA Opossum	South Louisiana	I	I	I	I	I
Raccoon	92122102R	Statesboro, GA	IV	IV	IV	IV	IV
(Carnivora)	93040701R cl2	Statesboro, GA	IV	IV	IV	IV	IV
	93053103R cl3	Harrold Preserve, GA	I	I	I	I	I
	93071502R cl2	Fort Stewart, GA	IV	IV	IV	IV	IV
	93072805R cl3	Fort Stewart, GA	IV	IV	IV	IV	IV
	FL Rac 13	Maclay State Park, FL	I/IV	I	I	IV	n.d.
	FL Rac 15	Wakulla Springs, FL	IV	IV	IV	IV	IV
	FL Rac 30	Wakulla Springs, FL	IV	IV	IV	IV	IV
	FL Rac 46	Tall Timbers, FL	IV	IV	IV	IV	n.d.
	FL Rac 5	Torreya State Park, FL	IV	IV	IV	IV	n.d.
	FL Rac 7	Lake Talquin, FL	IV	IV	IV	IV	IV
	FL Rac 9	Torreya State Park, FL	IV	IV	IV	IV	IV
	GA Rac 107	Ossabaw Island, GA	IV	IV	IV	IV	IV
	GA Rac 134	Whitehall Forest, GA	IV	IV	IV	IV	IV
	GA Rac 143	Athens, GA	IV	I	IV	IV	IV
	GA Rac 45	Skidaway Island, GA	IV	IV	IV	IV	n.d.
	GA Rac 69	Athens, GA	IV	IV	IV	IV	IV
	Maryland Rac	Laurel, MD	IV	IV	IV	IV	IV‡
	STC 10R cl3	St. Catherine's Island, GA	IV	IV	IV	IV	IV
	STC 35R	St. Catherine's Island, GA	IV	IV	IV	IV	IV
	TN Rac 18	Rutherford Co., TN	IV	IV	IV	IV	IV
*T. sanguisuga*	Florida	Gainesville, FL	I	I	I	I	n.d.
(Hemiptera)	Florida C1F8	Gainesville, FL	I	I	I	I	IV
	T. sang 5 cl1	Bulloch Co., GA	I	IV	IV	IV	n.d.
RT lemur	Nilda	St. Catherine's Island, GA	IV	IV	n.d.	IV	n.d.
(Primate)	Clarence	St. Catherine's Island, GA	IV	IV	IV	IV	IV‡
	Meg	St. Catherine's Island, GA	IV	IV	IV	n.d.	IV‡
Rh. Macaque (Primate)	Texas Theis	Not known	I	IV	IV	n.d.	IV
Nb Armadillo (Cingulata)	Armadillo 1973	New Orleans, LA	I	I	I	I	I
	GA Arm 20	Ossabaw Island, GA	IV	IV	IV	IV	IV
	USA Armadillo	South Louisiana	I	I	I	I	I
Str. Skunk (Carnivora)	GA Sk 1	Ludiwici, GA	IV	IV	IV	n.d.	IV‡

* Table abbreviations: VA opossum = Virginia Opossum; RT lemur = Ring-tailed Lemur; Rh. Macaque = Rhesus Macaque; Nb Armadillo = Nine-banded Armadillo; Str. Skunk = Striped Skunk; n.d. = not determined.

† previously characterized using mini-exon, D7 divergent domain of 24 s alpha rRNA, and 18 s rRNA genetic analysis in [34].

‡ partial sequences were analyzed.

### Molecular Technique

Template was obtained for polymerase chain reactions by boiling parasites for 15 min and using the resulting supernatant for DNA extraction with the DNeasy blood and tissue kit (Qiagen, Inc., Valencia, CA) following the manufacturer's protocol. PCR amplification with GoTaq *Taq* polymerase (Promega Corporation, Madison, WI) was completed for the four gene targets, *MSH2*, *Tc52*, *DHFR-TS*, and *COII-ND1*, following the respective previously published protocols [27], [28], [10]. DNA extraction, amplification, and product analysis were performed in separate dedicated laboratory areas. A negative water control was included in each set of extractions and PCR reactions as contamination controls. Sequencing reactions were performed at the Clemson University Genomics Institute (Clemson, SC). Sanger sequencing reactions were carried out with purified PCR product and amplicons were bidirectionally sequenced on an ABI 3100 Automated Sequencer using the provided ABI equipment software for basecalling and sequencing analysis (Applied Biosystems, Foster City, CA). In the case of *COII-ND1* products, reactions that did not yield complete sequences were identified and a heminested PCR reaction was performed using primers ND1.3A and COII.2A in the primary reaction and ND1.3S and COII.A400 or COII.2A and COII.A400R in the secondary reactions [10]. Because of the size (1,473 bp, *DHFR-TS*; 1,226 bp, *COII-ND1*; 875 bp, MSH2; 1,300 bp, Tc52) and inherent properties of the amplicons, cloning attempts were unsuccessful. Therefore, multiple sequences were obtained for each isolate and polymorphisms were noted if observed in at least two sequences for an analyzed sample.

### Phylogenetic Analysis and Genotyping

Contiguous sequences were assembled in Sequencher and sequences aligned by the Clustal Wallis method in Mega4. Three phylogenetic trees were created by neighbor-joining, minimum evolution and maximum parsimony methods from the alignment of each gene target with the bootstrap consensus tree being inferred from 500 replicates and the bat trypanosome *T. cruzi marinkellei* [593 (B3)] or *T. brucei* [TReu927] used as the outgroup [29]–[32]. A strict consensus tree was interpreted from the three methods based on topology with no observed bootstrap supported incongruencies. Evolutionary distances were computed using the Kimura 2-parameter method [33]. Lineage typing of each isolate was performed with whole or partial sequences of the obtained gene sequence and a BLAST search was administered on GenBank to determine sequence identity with previously genotyped *T.* cruzi strains. Nucleotide sequence data reported in this paper are available in the GenBank database under accession numbers: GU212870-GU212990, GU212992-GU213035.

## Results

Based on *MSH2*, *Tc52*, and *DHFR-TS* sequences, all human (TcI), ring-tailed lemurs (TcIV), armadillo (TcI or TcIV) and skunk (TcIV) isolates were genotyped as the equivalent lineages previously determined [34]. In contrast, some isolates from domestic dogs, Virginia opossums, raccoons, a rhesus macaque, and a *Triatoma sanguisuga* were classified as different lineages by different gene targets. The domestic dog isolate ‘Griffin Dog,’ previously thought to be a mixed population of TcI and TcIV [34], was confirmed in this study to have multiple sequences consistent with the TcI and TcIV reference isolates. A mixed population was demonstrated with polymorphic positions identified in the *MSH2* and *Tc52* genes (Tables 2 and 3).

**Table 2:** Nucleotide sequence variations within the MSH2 gene sequence of 50 *T. cruzi* isolates from the United States compared to reference strains.

Genotype/Isolate	Nucleotide Position																				
	71	97	109	172	244	351	367	373	403	404	460	478	490	500	634	645	658	735	750	775	816
*TcI Reference strain (Silvio X10 cl1)*	C	A	G	A	C	T	A	G	G	G	A	T	C	A	T	C	A	C	A	G	A
TcI – 17 US sequences*	•	•	•	•	•	A	•	•	•	•	•	•	•	•	•	•	•	•	•	•	•
TcI-USA Opossum	•	•	•	•	•	A	•	•	•	•	•	•	•	•	•	•	•	•	•	•	G
TcI-93070103P cl2	•	•	•	•	•	A	•	•	•	•	•	Y	•	•	Y	S	•	•	T	•	•
Amino acid change						V→D										S→C					

*TcIV Reference strain (CANIII cl1)*	G	G	G	G	T	A	G	A	G	G	A	C	T	C	C	G	A	C	A	G	A
TcIIa-24 US sequences†	•	A	•	•	•	•	•	G	•	•	T	•	C	•	•	•	•	•	•	•	•
TcIIa-TN Rac 18	•	A	•	•	•	•	•	G	A	A	T	•	C	M	•	•	•	•	•	•	•
TcIIa-STC 10R cl3	•	A	•	•	•	•	•	G	•	•	T	•	C	•	•	•	G	•	•	•	•
TcIIa-Fl Rac 7	•	A	•	•	•	•	•	G	•	•	T	•	C	•	•	•	•	G	•	•	•
TcIIa-GA Rac 107	•	A	•	•	•	•	•	G	•	•	T	•	C	•	•	•	•	•	•	A	•
TcIIa-FL Rac 30	•	A	•	•	•	•	•	G	•	•	T	•	C	•	•	•	T	•	•	•	•
TcIIa-T. sang 5 cl1	•	A	T	•	•	•	•	G	•	•	T	•	C	•	•	•	•	•	•	•	•
TcI/IIa-Griffin Dog	•	A	•	•	Y	A	R	G	•	•	W	Y	C	M	Y	S	•	•	•	•	•
Amino acid change										V→M				I→L				T→R			

Genotype of each isolate precedes the isolate name. Nucleotide positions correspond to sites from SilvioX10 cl1 (Genbank AY540739). Dots represent nucleotide site identical to reference strain (either Silvio X10 cl1 for TcI or CANIII cl1 for TcIV).

* The following 17 US TcI sequences were identical: Human isolates (TC CC, CA R, TC California), domestic dogs (USA Dog Y), Virginia opossums (92101601P cl2, 93041401P cl2, FH4, FL Opo 2, FL Opo 3, FL Opo 15, Opossum 1970), armadillos (Armadillo 1973, USA Armadillo), triatomine bugs (Florida C1F8, Florida), and raccoons (93053103R cl3, FL Rac 13).

† The following 23 US TcIV sequences were identical: Raccoons (FL Rac 9, 92122102R, 93071502R cl2, 93040701R cl1, 93072805R cl3, FL Rac 15, FL Rac 46, FL Rac 5, GA Rac 134, GA Rac 143, GA Rac 69, Maryland Rac, STC 35R), domestic dogs (Samantha Dog, Caesar Dog, Dog Theis, OK Dog, Smokey), ring-tailed lemurs (Clarence, Meg), rhesus macaque (Texas Theis), striped skunk (GA Sk 1), and armadillo (GA Arm 20).

**Table 3:** Nucleotide sequence variations within the Tc52 gene sequence of 51 *T. cruzi* isolates from the United States compared to reference strains.

Genotype/Isolate	Nucleotide Position																	
	85	91	121	148	151–153	155–156	159–160	200	221	231	242	336	357	392	443	500	560	588
*TcI reference strain (P209)*	C	A	T	G	ACA	GG	CT	A	A	T	T	A	C	T	G	A	G	A
TcI – 17 US sequences*	•	•	•	•	•••	••	••	•	•	•	•	•	•	•	•	•	•	•
TcI-TC California	•	•	•	•	•••	••	••	•	•	•	•	•	•	•	•	•	•	•
TcI-93041401P cl1	•	•	C	•	•••	••	••	•	•	•	•	•	•	•	•	•	•	•
TcI-FL Opo 2	•	•	•	•	•••	••	••	•	•	•	•	•	•	•	•	•	•	•
Amino acid change			V→A				L→P											
***TcIV reference strain (CANIII cl1)***	**A**	**A**	**T**	**G**	**ACA**	**GG**	**CT**	**G**	**A**	**T**	**C**	**G**	**G**	**C**	**G**	**G**	**G**	**G**
TcIIa- 27 US sequences†	C	•	•	•	•••	••	••	A	G	G	T	•	•	•	A	•	•	A
TcIIa-TN Rac 18	C	C	•	A	GGT	CA	GA	A	G	G	T	•	•	•	A	•	•	A
TcIIa-FL Rac 7	C	•	•	•	•••	••	••	A	G	G	T	•	•	•	A	•	A	A
TcI/IIa-Texas Theis	•	•	•	•	•••	••	••	A	G	G	•	•	•	•	A	•	•	A
TcI/IIa-Griffin Dog	•	•	•	•	•••	••	••	A	R	K	•	K	S	Y	R	R	•	A
Amino acid change	E→A	E→A		Y→W	K→Y	E→K	L→E			S→A								A→T

Genotype/Isolate	Nucleotide Position																						
	603	621	625	628	659	670	728	816	852	873	875–876	920	967–968	971	980	1021	1048	1103	1140	1143	1251	1262	1295
*TcI reference strain (P209)*	A	G	T	A	A	C	C	T	G	A	CC	A	TA	T	C	T	T	C	G	T	A	A	A
TcI – 17 US sequences*	•	•	•	•	•	•	•	•	•	•	••	G	••	•	•	•	G	•	•	•	•	G	•
TcI-TC California	•	•	•	•	•	•	•	•	•	•	••	•	••	•	•	•	•	•	•	•	•	•	•
TcI-93041401P cl1	•	•	•	•	•	•	•	•	•	•	••	G	••	•	•	•	G	•	•	•	•	G	•
TcI-FL Opo 2	•	•	•	•	•	A	•	•	•	•	••	G	••	•	•	•	G	•	•	•	•	G	•
Amino acid change						T→K											R→M						
***TcIV reference strain (CANIII cl1)***	**G**	**A**	**C**	**G**	**T**	**A**	**T**	**G**	**A**	**G**	**AT**	**G**	**CG**	**C**	**G**	**A**	**T**	**G**	**G**	**A**	**G**	**G**	**G**
TcIIa- 27 US sequences†	A	•	•	A	•	•	•	T	•	A	C•	•	••	•	•	•	•	•	A	•	•	•	•
TcIIa-TN Rac 18	A	•	•	A	•	•	•	T	•	A	C•	•	••	•	•	•	•	•	A	•	•	•	•
TcIIa-FL Rac 7	A	•	•	A	•	•	•	T	•	A	C•	•	••	•	•	•	•	•	A	•	•	•	•
TcI/IIa-Texas Theis	A	•	•	A	•	•	•	T	•	A	C•	•	••	•	•	•	•	•	A	•	•	•	•
TcI/IIa-Griffin Dog	A	•	Y	A	W	M	Y	T	R	A	CY	•	YR	Y	S	W	K	S	R	W	-	-	-
Amino acid change	A→T		L→S	R→K				G→W		A→T									A→T				

Genotype of each isolate precedes the isolate name. Nucleotide positions correspond to sites from P209 (Genbank EF065175). Dots represent nucleotide site identical to reference strain (either P209 for TcI or CANIII cl1 for TcIV). Dashes represent missing nucleotides.

* The following 17 US TcI sequences were identical: Human isolates (TC CC, CA R), domestic dogs (USA Dog Y), Virginia opossums (USA Opossum, Opossum 1970, 92101601P cl2, FH4, 93070103P cl2, FL Opo 3, FL Opo 15), armadillos (Armadillo 1973, USA Armadillo), triatomine bugs (Florida C1F8, Florida), and raccoons (GA Rac 143, 93053103R cl3, FL Rac 13).

† The following 26 US TcIV sequences were identical: Raccoons (STC 10R cl3, FL Rac 9, 92122102R, 93071502R cl2, 93040701R cl1, 93072805R cl3, FL Rac 15, FL Rac 46, FL Rac 5, FL Rac 30, GA Rac 134, GA Rac 69, GA Rac 107, Maryland Rac, STC 35R), domestic dogs (Samantha Dog, Caesar Dog, Dog Theis, OK Dog, Smokey), ring-tailed lemurs (Clarence, Meg, Nilda), striped skunk (GA Sk 1), triatomine bug (T sang5 cl1), and armadillo (GA Arm 20).

**Figure 1:**
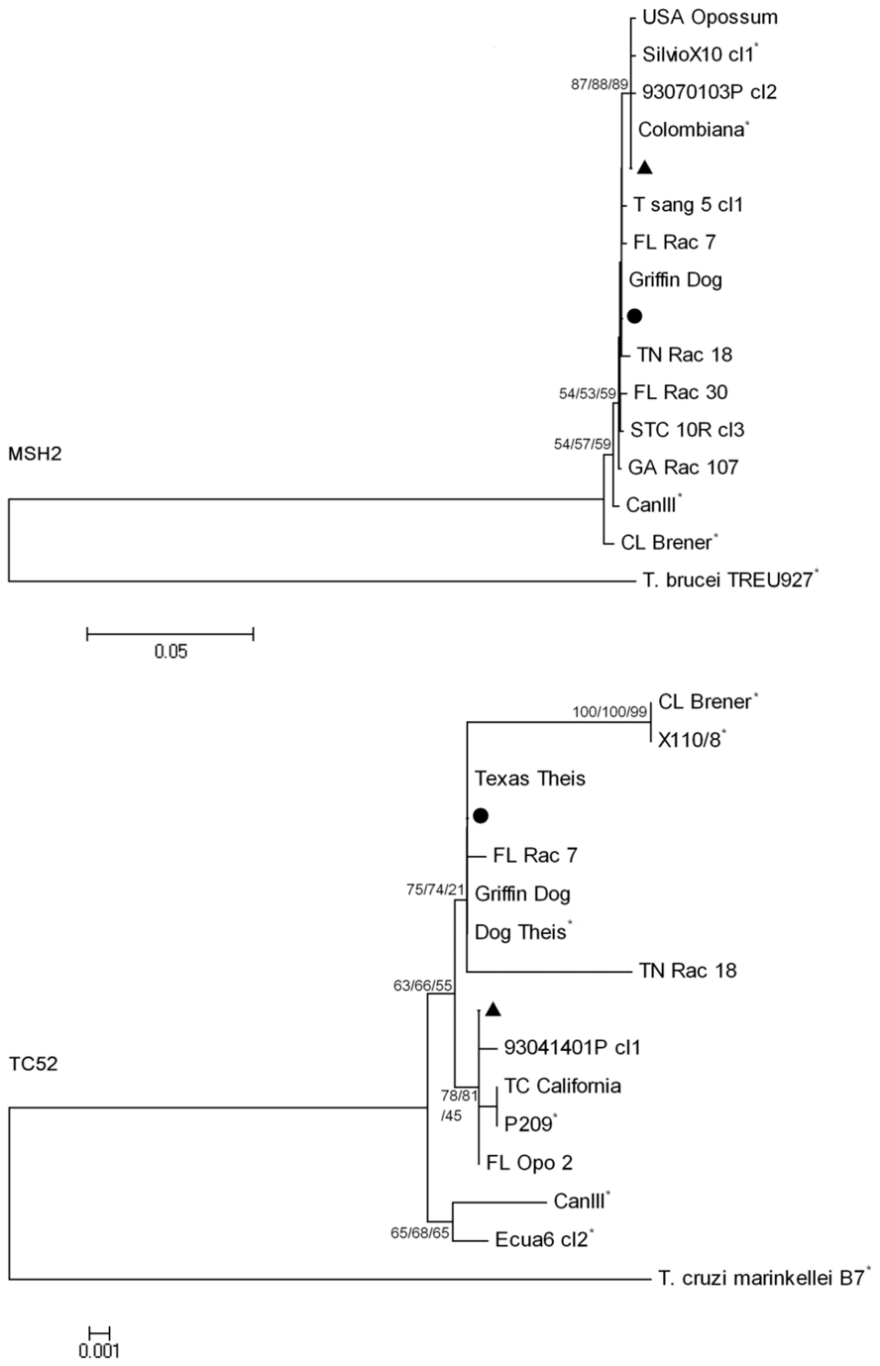
Evolutionary relationships among mismatch-repair class 2 gene (*MSH2*) and the thiol-disulfide oxido-reductase Tc52 gene (*Tc52*) from 50 and 51 *Trypanosoma cruzi* isolates, respectively. Three phylogenetic trees were created by neighbor-joining (NJ), minimum evolution (ME), and maximum parsimony (MP) methods from the alignment of each gene target and a consensus tree was interpreted. Numbers at the branches are bootstrap values >50% (500 replicates) for the same nodes of the NJ, ME, MP trees. Evolutionary distances were computed using the Kimura 2-parameter method [29]. ▴ = the 17 US TcI isolates that were identical; • = the 24 or 27 US TcIIa isolates that were identical. * = reference strains: SilvioX10 cl1, Colombiana, P209 (TcI); X110/8 (TcIII); CANIII cl1, Dog Theis, Ecua6 (TcIV); CL Brener (TcVI).

Single nucleotide polymorphisms (SNPs) were observed in the *MSH2* and *Tc52* genes of the analyzed sequences compared to TcI and TcIV reference strains from South America (Tables 2 and 3). The majority of sequences for the *MSH2* (19TcI and 24 TcIV) and *Tc52* (18 TcI and 27 TcIV) genes were identical among the US isolates (Tables 2 and 3). For the *MSH2* gene, only one or two nucleotides distinguished US TcI isolates from the reference strain (Silvio X10 cl1) while four to six nucleotides distinguished US TcIV from the reference strain (CANIII cl1) (Table 2). For the *Tc52* gene, three to four nucleotide substitutions distinguished the US TcI isolates from the reference strain (P209) with one exception; a human isolate from a California patient (Tc California) was identical to the South American reference strain (Table 3). Numerous SNPs [13]–[22] distinguished the Tc52 sequences of US and reference TcIV strains (CANIII cl1). Overall at least four SNPs were identified in these two genes that could be used to separate US isolates of TcI and TcIV from the two South American reference strains (Tables 2 and 3). Several nucleotide changes resulted in amino acid changes (Tables 2 and 3). The phylogenies of the two gene targets show the clustering of isolates with their respective genotype, including those that exhibited unique SNPs (Figure 1).

**Figure 2:**
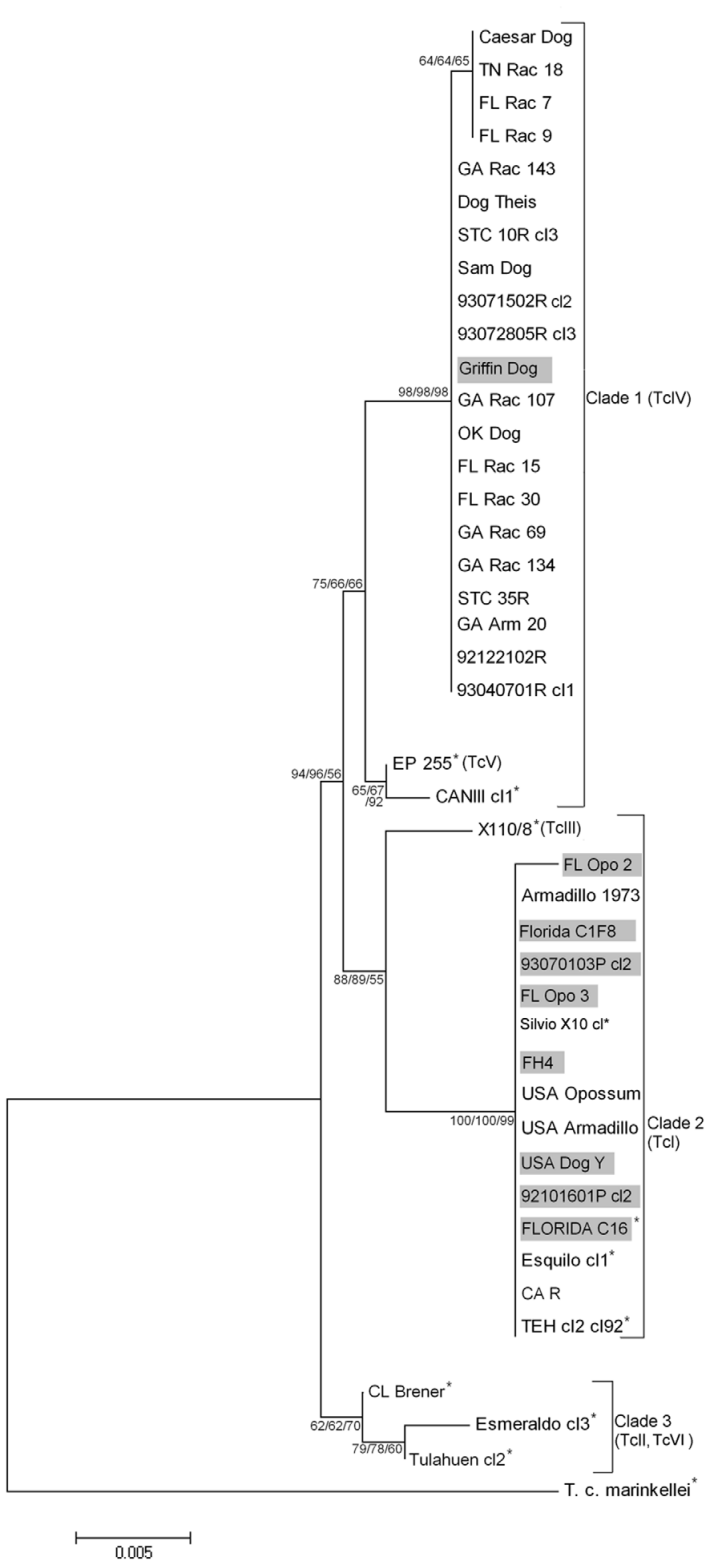
Evolutionary relationships among dihydrofolate reductase-thymidylate synthase (*DHFR-TS*) from 43 *Trypanosoma cruzi* isolates. Three phylogenetic trees were created by neighbor-joining (NJ), minimum evolution (ME), and maximum parsimony (MP) methods from the alignment of each gene target and a consensus tree was interpreted. Numbers at the branches are bootstrap values >50% (500 replicates) for the same nodes of the NJ, ME, MP trees. Evolutionary distances were computed using the Kimura 2-parameter method [29]. The nine isolates with positions incongruent to the mitochondrial phylogenies (Fig. 3) are highlighted. * = reference strains. Sequences clustered in 3 clades: Clade 1 includes TcIV *T. cruzi* isolates from the US and reference TcIV and TcV S. America strains. *T. cruzi* isolates of TcI lineage from the US and reference strains clustered in Clade 2, while Clade 3 consists on TcII and TcVI S. American reference strains.

Phylogenetic analysis of the nuclear gene region, *DHFR-TS*, supported the findings of the *Tc52* and *MSH2* gene analyses and resulted in a tree that had a similar topology to a previous study [10] with three major clades (Figure 2). Little diversity was present between US TcIV isolates, but all US TcIV sequences branched separately from the reference South American TcIV sequence. Similar to sequence analysis results for the *DHRF-TS* gene of various TcI isolates [10], limited differences were noted within the US isolates as no separation of TcI sequences was present (Figure 2). TcIV isolates had 99% sequence identity to the CANIII cl1 reference strains with 7 SNPs; TcI isolates had 99% sequence identity to the Silvio X10 cl1 reference strain with 4 SNPs.

**Figure 3:**
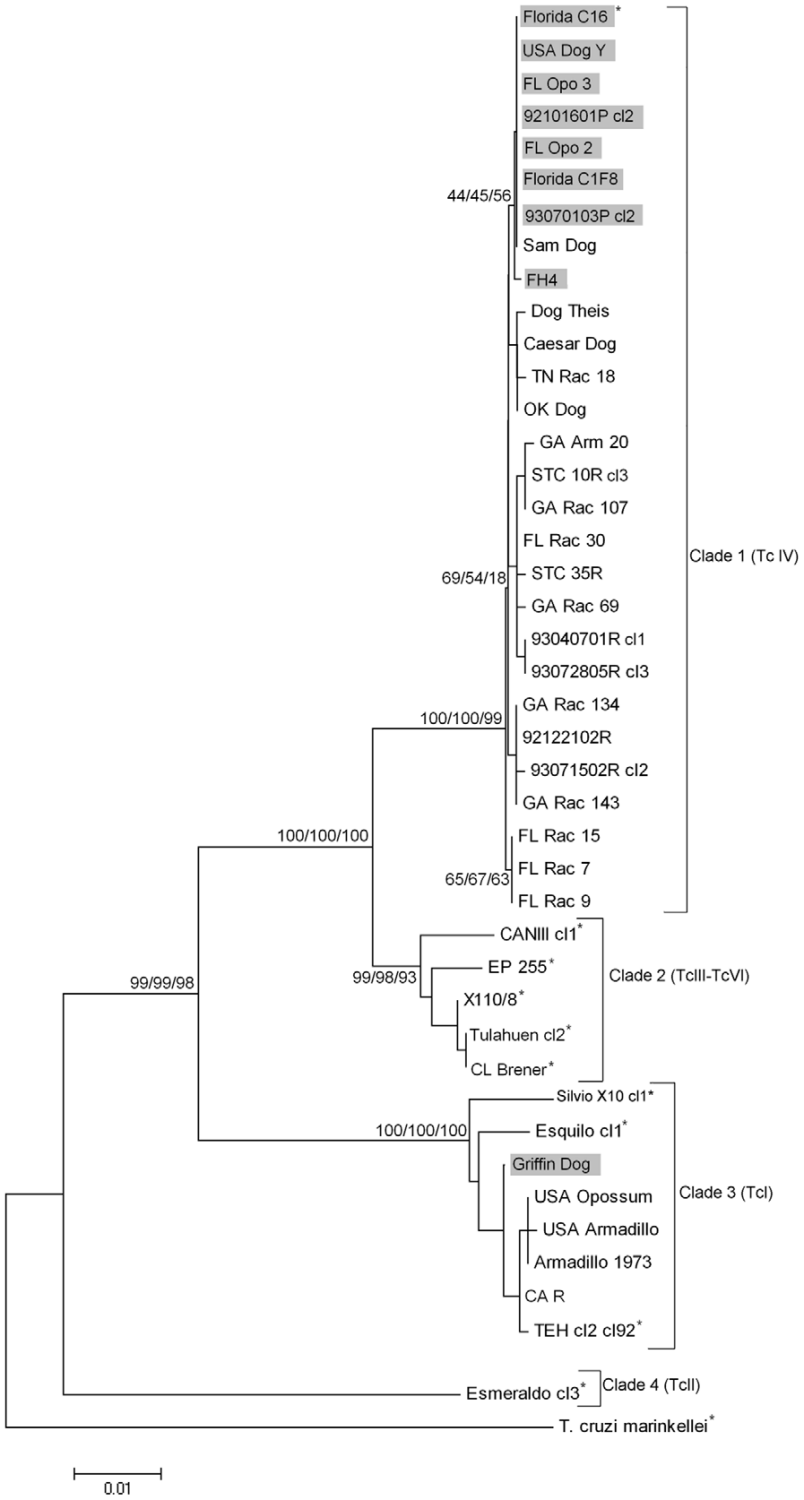
Evolutionary relationships among cytochrome oxidase subunit II- NADH dehydrogenase subunit I region (*COII-ND1*) from 43 *Trypanosoma cruzi* isolates. Three phylogenetic trees were created by neighbor-joining (NJ), minimum evolution (ME), and maximum parsimony (MP) methods from the alignment of each gene target and a consensus tree was interpreted. Numbers at the branches are bootstrap values >50% (500 replicates) for the same nodes of the NJ, ME, MP trees. Evolutionary distances were computed using the Kimura 2-parameter method [29]. The nine isolates with positions incongruent to the nuclear phylogenies (Fig. 2) are highlighted. * = reference strains. Sequences clustered in 4 distinct clades. Clade 1 contains exclusively US origin TcIV strains of *T. cruzi*. Reference TcIII-TcIV strains of *T. cruzi* clustered in Clade 2. TcI *T. cruzi* from the US and S. American reference strains clustered in Clade 3, while a separation of a TcII reference strain results in the fourth clade.

The phylogeny for the *COII-ND1* mitochondrial region showed greater divergence with four clades containing additional divisions (Figure 3). Eight isolates that were classified as TcI by analysis of various nuclear genes (e.g., 18S, mini-exon, 24S alpha, *MSH2*, *Tc52*, *DHFR-TS*) were classified as TcIV by phylogenetic analysis of *COII-NDI* sequences. A single TcIV isolate, Griffin Dog, had an incongruent phylogenetic position. These nine isolates are highlighted in Figures 2 and 3.

SNPs were not observed among the *COII-ND1* US TcIV isolate sequences, but they only had 96% sequence identity to the CANIII cl1 reference strain with 36 SNPs (data not shown). Among the *COII-ND1* US TcI isolate sequences, 88 SNPs were identified so that two TcI alleles were identified (data not shown). One allele corresponds to TcI strains proposed to exhibit evidence of genetic exchange based on the mitochondrial and nuclear incongruent phylogenies seen in Figures 2 and 3, while the other consists of TcI strains that do not show evidence of such events. Compared with the TcI reference strain, those that did exhibit exchange events had 92% sequence similarity to the Silvio X10 cl1 reference strain, with 85 SNPs; sequences without genetic exchange had 98% sequence identity to Silvio X10 cl1, with only 19 SNPs.

## Discussion

In the current investigation, genetic diversity was demonstrated among *T. cruzi* isolates from the United States. *T. cruzi* strains are currently categorized into six major lineages, TcI to TcVI and a Tcbat genotype [5]–[8]. All six major genotypes have been characterized from South American isolates from various host species [35]. Contrastingly, strains from Mexico and Central America (Guatemala) have been characterized as TcI (both) and TcIV (Guatemala only), with a clear predominance of TcI isolates [36]–[39]. Isolates from the United States, have also been characterized only as TcI or TcIV [34], [40], [41]. Further confirming the paucity of genotypes in North America, in the current study, sequences of additional gene targets had sequence identity only to either TcI or TcIV. Regardless of gene target, TcIV isolates were clearly distinguished from the South American TcIV reference strain which provides additional evidence for considerable divergence within this lineage [10], [42]–[44].

To investigate genetic diversity among US *T. cruzi* isolates, the sequences of two nuclear genes, *Tc52* and *MSH2*, were analyzed to identify SNPs. *Tc52* is a single-copy gene constitutively-expressed in all developmental stages of *T. cruzi* and is implemented in the immune response to *T. cruzi* infection, where it suppresses T-cell proliferation by scavenging cysteine and glutathione (GSH) [45], [46]. Similar to previous findings [28], [47], [42], numerous SNPs were found in the sequences of 50 isolates analyzed in this study. Of the 47 SNPs identified, 17 resulted in amino acid changes, several of which have been previously linked to GSH binding [28]. Additional research is needed to determine if there is an association of these SNPs with biological differences (e.g., virulence) due to changes in GSH binding efficacy or between isolates from the US and those from South America.

Polymorphisms were also identified in *MSH2*, a homologue of the *mutS* gene of other eukaryotes [48]. The MSH2 protein is a part of the mismatch repair machinery that binds base-base mismatches and excises and repairs them. In *T. cruzi*, *MSH2* is also a single copy gene that is constitutively-expressed in all life stages of the parasite [48]. In the current investigation, we identified 21 SNPs of the *MSH2* gene, including several that could distinguish between TcI and TcIV strains. Previous findings suggested that SNPs in TcII lineage had decreased mismatch-repair ability compared to TcI strains [27]. In our study, the majority of genetic variability was noted in the TcIV isolates. Interestingly, TcIV isolates from the US tend to be less virulent to laboratory mice and to date, no human infections with this genotype have been reported in North America [49]; TcIV strains have been isolated from primates, prosimians, and domestic dogs [34], [50], [51]. Previously, different *T. cruzi* MSH2 phenotypes have exhibited different levels of susceptibility to cisplatin and oxidative damage [48], [52]. The ability or inability to withstand such external pressures from DNA damaging compounds was associated with genetic variability and subsequent strain differences [48], [52]. One may speculate, then, that the sequence differences within and between lineages observed in the current study may result in phenotypic differences affecting drug susceptibility. In addition to identifying sequence differences in these US isolates, phylogenies were constructed for *DHFR-TS* (nuclear) and *COII-ND1* (mitochondrial) to elucidate genealogical relationships among isolates and illustrate evidence for genetic exchange. The nuclear phylogeny of *DHFR-TS* exhibited three major clades. Isolates of TcI from the US clustered with S. American isolates, illustrating the limited genetic variability of the lineage reported in previous studies with this gene [10], [39]. Although genetic variability among TcI isolates was minimal for nuclear gene targets in this study, considerable biological differences between isolates have been previously noted [53]–[55]. Other studies have differentiated TcI isolates using the microsatellite analysis, comparative genome hybridization, and mini-exon and cytochrome *b* genes, sometimes suggesting the subdivision of the lineage [13], [14], [56]–[59]. Division between N. and S. American TcIV strains may be evidence of the independent evolution of N. American TcIV strains from its ancestral S. American strains, as is supported by the results of this and previous studies [43], [60].

The phylogeny of *COII-ND1* demonstrated greater genetic diversity with additional clustering occurring within the four clades present. As with the nuclear phylogeny, TcIV strains from the US diverged from TcIV strains of S. America. Additional clusters within the US TcIV clade indicate additional genetic diversity within the group; however, several of these subclades had low bootstrap support. The clade representing TcI strains contained both US isolates from this study and South American isolates, which is consistent with the nuclear *DHFR-TS* phylogeny. As previously suggested, the clustering of all TcI sequences may be due to a single origin of these strains [39], [61]. It is also possible that TcI represents a more recent introduction or spread into North America compared with TcIV, which may have been separate from the South American strains for a significant period of time which would allow divergence.

The most compelling finding from the *COII-ND1* phylogeny is the clustering of several TcI strains (classified based on several nuclear genes) within the US TcIV clade. Incongruencies between nuclear and mitochondrial phylogenies have been previously reported with S. American isolates and a single US isolate and is interpreted as evidence of rare genetic exchange events in the *T. cruzi* population [10], [62]. These findings in addition to in vitro demonstration of genetic recombination and previous multilocus sequence typing studies illustrate that genetic exchange does occur, albeit rarely [10], [11], [43], [62]. In the current study, several TcI sequences represent isolates that may have undergone genetic exchange in comparison to few TcIV sequences. This suggests that TcI isolates may be more susceptible or likely to have recombination, possibly as a result of more rapid evolution in this lineage [61]. While these phylogenies can be associated with genetic exchange, the role of such events in driving the evolution of the species has not been explored [61]. In the current study, we identified several isolates with evidence of genetic exchange. While only two (TcI and TcIV) of the six genealogical lineages have been detected circulating in mammal populations in the US, the presence of SNPs and evidence of genetic exchange suggest that parasite populations in the US are genetically diverse.
